# The long noncoding RNA CASC2 functions as a competing endogenous RNA by sponging miR-18a in colorectal cancer

**DOI:** 10.1038/srep26524

**Published:** 2016-05-20

**Authors:** Guanli Huang, Xiaoli Wu, Shi Li, Xiaoqun Xu, Hua Zhu, Xiangjian Chen

**Affiliations:** 1 Department of Surgical Oncology, The First Affiliated Hospital of Wenzhou Medical University, Wenzhou, Zhejiang, P.R. China; 2 Department of gastroenterology, The First Affiliated Hospital of Wenzhou Medical University, Wenzhou, Zhejiang, P.R. China; 3 Department of Urology, The First Affiliated Hospital of Wenzhou Medical University, Wenzhou, Zhejiang, P.R. China; 4 Operating room, The First Affiliated Hospital of Wenzhou Medical University, Wenzhou, Zhejiang, P.R. China; 5 Department of Obstetrics and Gynecology The First Affiliated Hospital of Wenzhou Medical University, Wenzhou, Zhejiang, P.R. China; 6 Department of endoscopic surgery, The First Affiliated Hospital of Wenzhou Medical University, Wenzhou, Zhejiang, P.R. China

## Abstract

Recent evidence highlights the crucial regulatory roles of long noncoding RNAs (lncRNA) in tumor biology. In colorectal cancer (CRC), the expression of several lncRNAs is dysregulated and play essential roles in CRC tumorigenesis. However, the potential biological roles and regulatory mechanisms of the novel human lncRNA, CASC2 (cancer susceptibility candidate 2), in tumor biology are poorly understood. In this study, CASC2 expression was significantly decreased in CRC tissues and CRC cell lines, and decreased expression was significantly more frequent in patients with advanced tumor-node-metastasis stage disease (TNM III and IV) (*P* = 0.028). Further functional experiments indicate that CASC2 could directly upregulate *PIAS3* expression by functioning as a competing endogenous RNA (ceRNA) for miR-18a. This interactions leads to the de-repression of genes downstream of *STAT3* and consequentially inhibition of CRC cell proliferation and tumor growth *in vitro* and *in vivo* by extending the G_0_/G_1_-S phase transition. Taken together, these observations suggest CASC2 as a ceRNA plays an important role in CRC pathogenesis and may serve as a potential target for cancer diagnosis and treatment.

Colorectal cancer is a highly lethal cancer worldwide with an increasing incidence each year^1^,^2^. Despite the diagnosis and therapeutic advances of colorectal cancer, the overall survival rate of colorectal cancer patients remains poor. In the past decades, intensive investigations identified a variety of molecular markers for CRC characterization and prognosis^3^,^4^. Recently, evidence has suggested that various lncRNAs also act as modulators in carcinogenesis and the progression of human colorectal cancer and may serve as novel therapeutic targets^5^,^6^,^7^.

To date, based on next-generation transcriptome sequencing (RNA-Seq) methods of individual samples, sequence read data reveal that more than 90% of the human genome is transcribed to produce many thousands of long noncoding RNAs (lncRNAs, >200 nucleotides in length)^8^,^9^,^10^. As a novel class of regulatory genes, lncRNAs lack significant protein-coding ability and have ignited a new area of biological investigation. Evidence suggests that lncRNA play important roles in a wide range of cellular processes, such as X chromosome inactivation, splicing, imprinting, epigenetic control and gene transcription regulation^11^,^12^,^13^,^14^,^15^. Studies indicate that lncRNAs are frequently aberrantly expressed in diverse human diseases, including various cancers^11^,^16^,^17^,^18^,^19^,^20^. Similar to protein coding genes, steadily growing evidence has revealed a new mechanistic role of these RNA species as part of a posttranscriptional regulatory network in cancer biology^21^,^22^. A large number of lncRNAs may function by competing with endogenous RNA (ceRNA) through a common MRE (miRNA response elements) for miRNA targets, thereby preventing a single miRNA or multiple miRNAs from binding to their proper regulatory targets^23^,^24^. A growing body of evidence strongly suggests that several lncRNAs, such as PTENP1^25^, H19^26^, HOTAIR^27^, and CCAT1^28^, may function as ceRNAs, exerting essential roles in many biological processes.

The novel lncRNA gene Cancer Susceptibility Candidate 2 (CASC2) is located on chromosome 10 in humans and has been characterized as a tumor suppressor in human malignancies, such as glioma^29^ and endometrial cancer^30^. Although the dysregulated expression of CASC2 in cancer patients highlights its tumorigenic properties, the molecular mechanisms underlying CASC2-mediated tumorigenesis remain largely unknown.

In this study, we first assessed the levels of CASC2 in CRC tissues and cell lines. Further experiments were conducted to investigate the biological function of CASC2 with respect to colorectal cancer cell phenotypes *in vitro* and *in vivo*. Additionally, mechanistic analysis reveals that CASC2 might function as a ceRNA to regulate *PIAS3* expression by sponging miR-18a, thus playing a critical role in the pathobiology of human colorectal cancer.

## Results

### Expression of CASC2 in both CRC cell lines and CRC tissues

**Figure 1:**
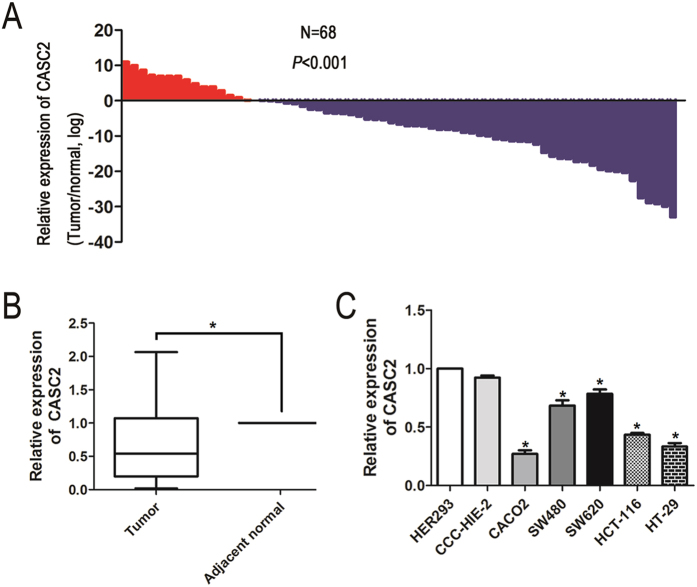
Analysis of CASC2 expression in both CRC tissues and cell lines. (**A**) CASC2 was significantly down-regulated in 76% (52/68) of CRC tissues. The blue shading indicates the down-regulation of CASC2, and the data are presented as fold-change in tumor tissues relative to normal tissues. *P* < 0.001, paired *t*-test. (**B**) Different CASC2 expression levels in CRC tissues and adjacent normal tissues from 68 patients as assessed by real-time PCR. *P < 0.05, paired *t*-test. (**C**) Relative expression of CASC2 in five CRC cell lines (CACO2, SW480, SW620, HCT116 and HT-29) and non-tumorigenic human cell lines (CCC-HIE-2 and HER293). *P < 0.05, Two-side Student’s *t*-test; n = 3.

The relative expression levels of CASC2 were measured using qRT-PCR in 5 CRC cell lines and a total of 68 patients with CRC, normalized to *GAPDH*. CASC2 was significantly down-regulated in 76% (52/68) of CRC tissues compared with adjacent normal CRC tissues (*P* < 0.001, Fig. 1A,B). We then evaluated whether CASC2 expression was associated with any clinicopathological parameters.

We divided the 68 patients with CRC into a high CASC2 tumor expression group (above the mean CASC2 expression, n = 34) and a low expression group (below the mean CASC2 expression, n = 34) (Table 1). As presented in Table 1, lower CASC2 expression was observed more frequently in patients with advanced tumor-node-metastasis stage (III and IV) (*P* = 0.028).

The level of CASC2 in CRC cell lines was further measured. Notably, all five CRC cell lines (CACO2, SW480, SW620, HCT-116 and HT-29) expressed lower levels of CASC2 than the non-tumorigenic cell lines (CCC-HIE-2 and HER293), especially CACO2 and HT-29 (Fig. 1C). The cell lines CACO2 and HT-29, which express relatively low CASC2 levels, were selected for further study to assess the potential functional role of CASC2.

Furthermore, another 80 paired CRC tissues and adjacent normal tissues were recently enrolled from the First Affiliated Hospital of Wenzhou Medical University to validate the expression of CASC2 using qRT-PCR. As expected, CASC2 expression was significantly down-regulated in cancerous tissues (*P* < 0.05; Supplementary Fig. 1A), and significantly correlated with TNM stage. Advanced TNM stage was correlated with decreased CASC2 expression (Supplementary Fig. 1B) indicating a suppressor role of CASC2 in CRC progression.

### Overexpression of CASC2 inhibits the proliferation of CRC cells *in vitro*

To assess the biological role of CASC2 in CRC, we first evaluated the impact of altered CASC2 expression in CRC cell lines.

**Figure 2:**
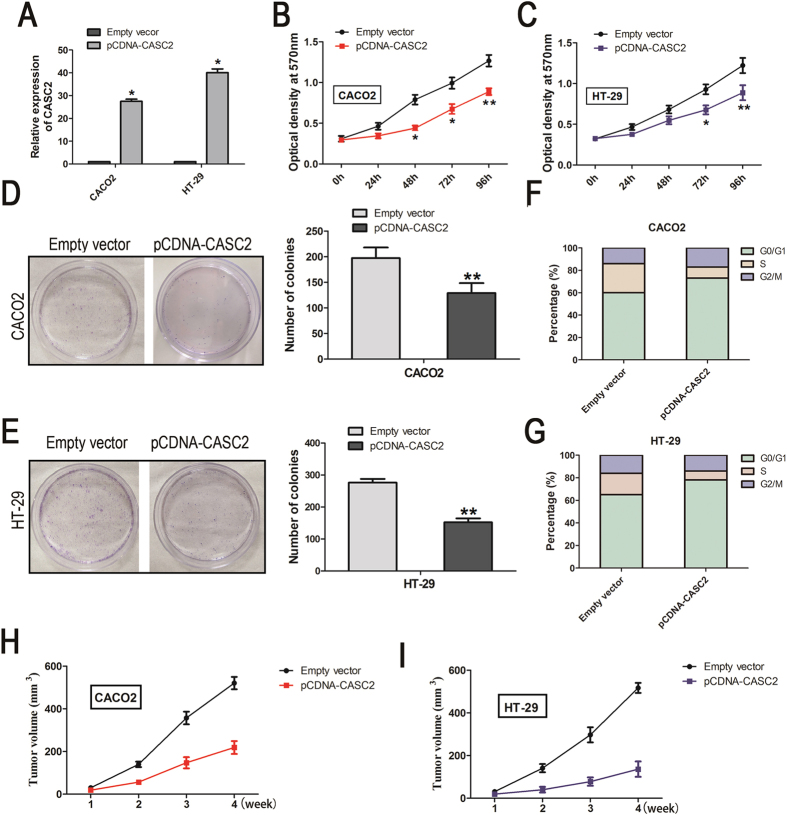
CASC2 overexpression suppressed CRC cells growth and tumor growth *in vitro* and *in vivo* by extending the G_0_/G_1_-S phase transition. (**A**) The relative expression level of CASC2 in CRC cell lines transfected with empty vector (control) or pcDNA-CASC2 was tested by qRT-PCR. *P < 0.05 were calculated by using the Two-sided Student’s *t*-test. CCK-8 assays were performed to measure the proliferation of CACO2 (**B**) and HT-29 cells (**C**). *P < 0.05 were calculated by using the Two-sided Student’s *t*-test method; n = 6. Colony-forming growth assays were performed to measure the proliferation of CACO2 (**D**) and HT-29 cells (**E**). *P < 0.05 were calculated by using the Two-sided Student’s *t*-test method; n = 3. Cell cycle analysis of CACO2 cells (**F**) and HT-29 cells (**G**) transfected with empty vector or pcDNA-CASC2. The data are presented as the mean ± SD from three experiments, *P < 0.05 compared with the control, Two-sided Student’s *t*-test; n = 3. The impact of CASC2 overexpression on tumor growth *in vivo* after CACO2 cells (**H**) and HT-29 cells (**I**) transfected with empty vector or pcDNA-CASC2. Tumor volumes were calculated after injection every 2 days. The experiment was repeated three times, and the data are presented as the mean ± SD (n = 6). *P < 0.05, Two-sided Student’s *t*-test.

Following transfection of pcDNA-CASC2, CASC2 expression was significantly increased 27-fold and 40-fold in the CACO2 and HT-29 cell lines, respectively, (Fig. 2A). Moreover, CCK8 and colony formation assays were also performed. CCK8 assays revealed that cell proliferation was significant decreased in the CACO2 and HT-29 cell lines transfected with pcDNA-CASC2 after 4 days in culture relative to the negative control (31% decrease, *P* < 0.01 for CACO2 cells; 33% decrease, *P* < 0.01 for HT-29 cells) (Fig. 2B,C). As expected, the numbers of CACO2 and HT-29 colonies formed were also significantly decreased by CASC2 overexpression (Fig. 2D,E).

Subsequently, cell cycle progression was further analyzed to examine the effect of CASC2 on CRC cell proliferation. As shown in Fig. 2F,G, compared with the negative control, the percentage of cells in the G0/G1 phase increased in CRC cells with ectopic CASC2 expression (60% to 79%, *P* < 0.05 for CACO2 cells and 65% to 78%, *P* < 0.05 for HT-29 cells). Taken together, the ability to suppress proliferation indicates that CASC2 may have the potential to act as a tumor suppressor.

### Inhibition of tumor growth by CASC2 overexpression

Finally, we explored whether high levels of CASC2 could inhibit tumor growth *in vivo*. CRC cells transfected with pcDNA-CASC2 or pcDNA-NC were inoculated into nude mice. As shown in Fig. 2H,I, four weeks after injection, tumors derived from the pcDNA-CASC2 group were dramatically smaller than the control tumors (218.52 ± 67.23 mm^3^ *versus* 520.71 ± 65.22 mm^3^, *P* < 0.01 for CACO2 cells; and 136.06 ± 80.42 mm^3^ *versus* 517.26 ± 52.48 mm^3^, *P* < 0.01 for HT-29 cells).

### CASC2 is a target of miR-18a

**Figure 3:**
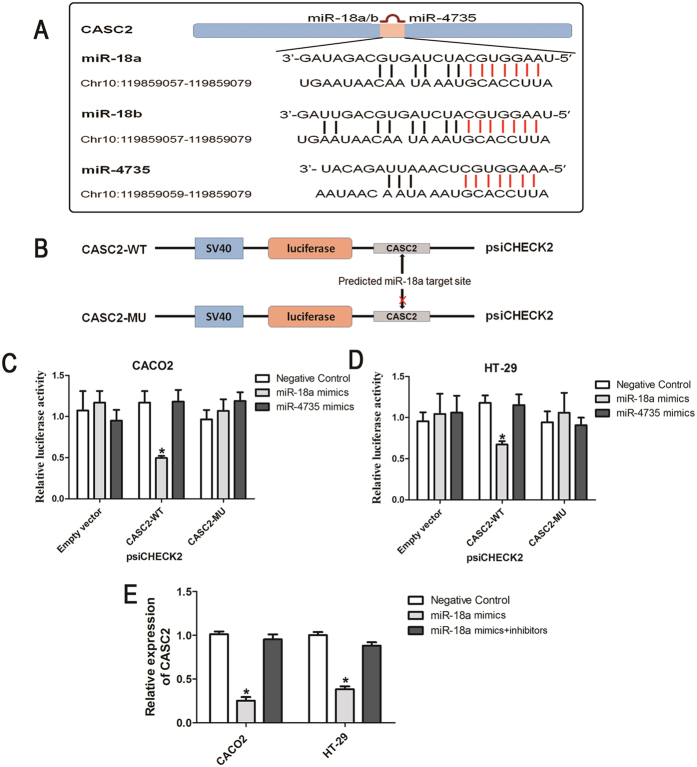
CASC2 functions as a miR-18a decoy in CRC cells. (**A**) The predicted positions of miR-18a/b and miR-4735 binding sites on the CASC2 transcript. (**B**) Schematic diagrams of two luciferase reporters containing the wild type CASC2 (psiCHECK2-CASC2-WT) and mutant CASC2 (psiCHECK2-CASC2-MU) abolishing targeting by miR-18a. Luciferase activity in CACO2 (**C**) and HT-29 (**D**) cells co-transfected with miR-18a or miR-4735 mimics and the indicated luciferase reporters or control. Renilla luciferase activity was measured and normalized to firefly luciferase. The experiment was repeated at least three times, and data are presented as the mean ± SD (*P < 0.05, Two-sided Student’s *t*-test; n = 3). (**E**) qRT-PCR for CASC2 levels in CRC cell lines transfected with miR-18a mimics, inhibitors or negative control (*P < 0.05; Two-sided Student’s *t*-test; n = 3).

Recently, a range of structural and functional classes of lncRNA sharing MREs have been reported to act as decoys to sequester miRNAs to prevent them from binding to targets and hence to modulate many functional mRNA targets through translation. Analysis with bioinformatics tools (webserver lnCeDB http://gyanxet-beta.com/lncedb/) that predict potential lncRNA-miRNA interactions revealed three potential CASC2 binding miRNAs (miR-18a/b and miR-4735) (Fig. 3A).

Interestingly, miR-18a and miR-18b belong to the miR-18 miRNA family and possess similar seed regions. Here, we primarily focused on miR-18a to further investigate the interaction between CASC2 and miR-18a in CRC cells. The wild type CASC2 (psiCHECK2-CASC2-WT) and mutant CASC2 constructs (psiCHECK2-CASC2-MU) were cloned downstream of the luciferase gene (Fig. 3B) and transfected in CRC cells together with either miR-18a or miR-4735 mimics.

Only the predicted miR-18a bound the CASC2 fragment containing the target sites of the miRNA. This resulted in 48% and 31% decrease in luciferase activity in CACO2 and HT-29 cell lines, respectively, compared with the empty vector control (Fig. 3C,D). The suppressive effects were successfully abolished when empty vector or mutant CASC2 reporter constructs for miR-18a were utilized, suggesting the binding of miR-18a to these sites. Subsequent qRT-PCR analysis revealed that CASC2 expression in CRC cells transfected with miR-18a mimics is suppressed by miR-18a (Fig. 3E). Together, these data indicate that miRNA-18a can directly bind to CASC2.

### miR-18a functions as an oncogene in CRC

**Figure 4:**
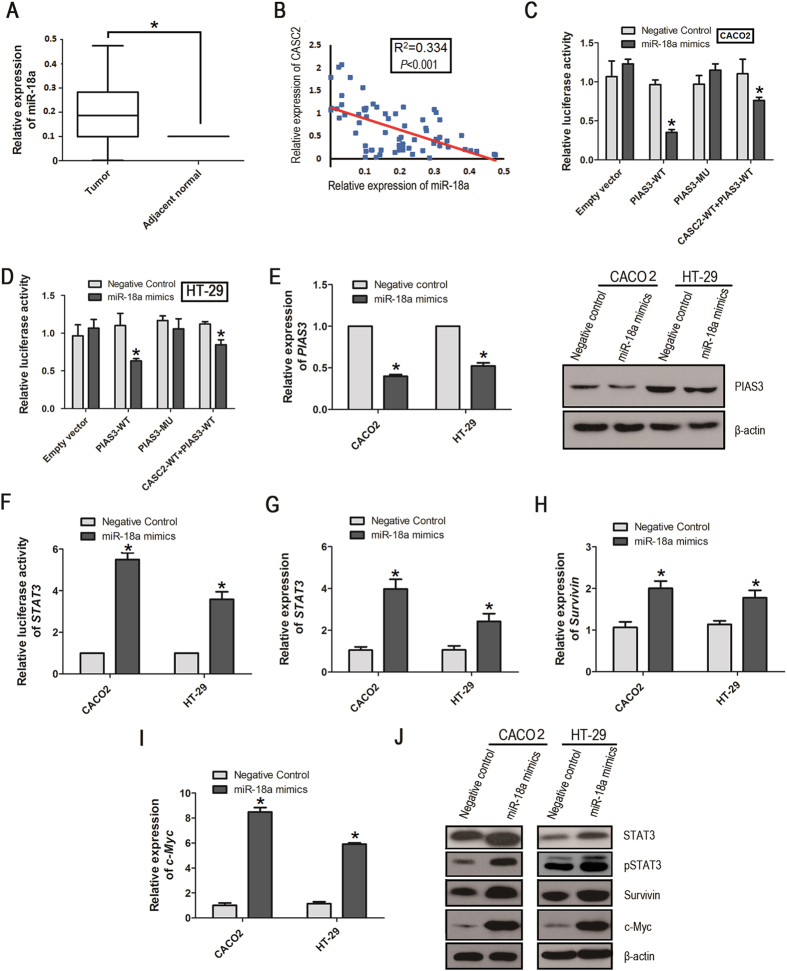
The expression of miR-18a in CRC, and the regulation of miR-18a on the *PIAS3* gene and STAT3 signaling pathway. (**A**) Relative miR-18a expression levels were examined by qRT-PCR in 68 CRC tissues and adjacent normal tissues. *P < 0.05, paired *t*-test. (**B**) The linear correlations between the expression levels of CASC2 and miR-18a in CRC tissues (R^2^ = 0.334, *P* < 0.001). The data were obtained using the logistic regression analysis. The luciferase reporter plasmids (RLuc-*PIAS3*-WT and RLuc-*PIAS3*-MT) containing the wild type 3′UTR region or mutant 3′UTR region of *PIAS3* were co-transfected into CACO2 cells (**C**) and HT-29 cells (**D**) with miR-18a mimics or in parallel with the luciferase reporter vector psiCHECK2-CASC2-WT. Error bars are representative of Mean ± SD (n = 3). *P < 0.05 was calculated using Two-side Student’s *t*-test. (**E**) miR-18a was transfected into CRC cell lines, and the mRNA and protein levels of *PIAS3* were evaluated by qRT-PCR (left) and western blot (right) 48 hours after transfection. β-actin was used as a loading control. (**F**) Luciferase reporter assay was performed to examine the effect of miR-18a on STAT3 transcriptional activity in CRC cells treated with miR-18a or negative control. Data are mean ± SD (n = 3). *P < 0.05, Two-side Student’s *t*-test. QRT-PCR analysis of *STAT3* (**G**), *Survivin* (**H**) and *c-Myc* (**I**) expression in CACO2 and HT-29 cells transfected with miR-18a mimics or negative control. The data are presented as the mean ± SD (n = 3), *P < 0.05; Two-sided Student’s *t*-test. (**J**) Western blot analysis was conducted to evaluate the effect of miR-18a on the regulation of the STAT3 signaling pathway, including STAT3, pSTAT3, Survivin and c-Myc.

The above results implicated the potential role of miR-18a within human tumor development, and we further determined the level of miR-18a expression in 68 paired CRC tissues and adjacent normal tissues. In contrast to CASC2, miR-18a expression is up-regulated in CRC tissues relative to adjacent normal controls (*P* < 0.001; Fig. 4A), which is consistent with previous studies^31^,^32^. Additionally, we investigated the relationship between miR-18a and CASC2. As predicted, there was a significant negative correlation in the expression of CASC2 and miR-18a in CRC tissues (R^2^ = 0.334, *P* < 0.001, Fig. 4B).

### miR-18a directly targets the ***PIAS3*** gene

Among the many targets of miR-18a, we concentrated on *PIAS3*^33^. Although the interaction between miR-18a and *PIAS3* has been predicted by computational algorithms and confirmed by functional experiments in gastric cells, it is unknown whether miR-18a effectively regulates *PIAS3* in CRC cells.

To test this, we constructed reporter plasmids by cloning the wild type 3′UTR region or mutant 3′UTR region of *PIAS3* downstream of the firefly luciferase reporter (RLuc-*PIAS3*-WT and RLuc-*PIAS3*-MT). The constructs were then transfected into CRC cells together with miR-18a for the luciferase reporter assay. Co-transfection of RLuc-*PIAS3*-WT and miR-18a mimics resulted in a 60% and 40% decrease in the luciferase signal in CACO2 (Fig. 4C) and HT-29 (Fig. 4D) cell lines, respectively, compared with the mutant vector or negative control. Next, we utilized qRT-PCR and western blot analysis to reveal that the ectopic expression of miR-18a significantly suppressed the mRNA and protein level of *PIAS3* in CRC cell lines (Fig. 4E), indicating that miR-18a can directly target *PIAS3*.

*PIAS3* was originally identified as a specific repressor of STAT3 signaling, and the altered STAT3 activity eventually contributes to cell growth and proliferation by promoting the expression of cell proliferation-associated proteins, such as Bcl-xL, Bcl-2, cyclin D1, Survivin, and c-Myc^34^,^35^.

To confirm the effect of miR-18a on the STAT3 signaling pathway, the STAT3 luciferase assay was employed to measure the transcriptional activity of STAT3 in CRC cells treated with miR-18a. As shown in Fig. 4F, the luciferase activity was significantly increased in CRC cells following transfection with the miR-18a mimics compared with cells transfected with the negative control. Additionally, qRT-PCR analysis and western blot analysis were further performed, and the miR-18a-treated cells were found to exhibit increased levels of STAT3, pSTAT3, Survivin and c-Myc (Fig. 4G–J). Thus, these findings demonstrate that overexpression of miR-18a may contribute to the downregulated expression of *PIAS3* and consequentially may enhance the expression of several STAT3-mediated genes.

### CASC2 modulates the level of the miR-18a target ***PIAS3***

**Figure 5:**
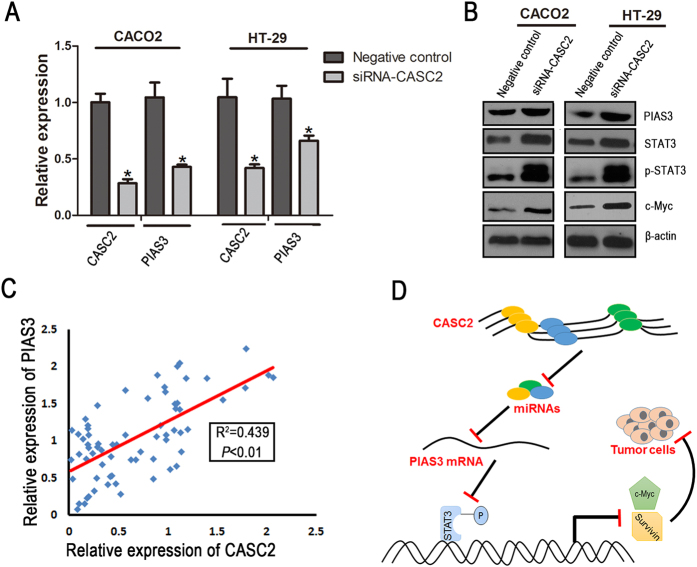
CASC2 functions as a ceRNA by competitively binding *PIAS3*-targeting miR-18a. (**A**) The expression of CASC2 and *PIAS3* mRNA in CACO2 and HT-29 cells treated with CASC2-siRNA or negative control. The data are presented as the mean ± SD (n = 3), *P < 0.05; Two-sided Student’s *t*-test. (**B**) Representative western blot of PIAS3, STAT3, pSTAT3 and c-Myc from CRC cell lines transfected with CASC2-siRNA or negative control. (**C**) The relationship between CASC2 and *PIAS3* in 68 CRC tissues (*P* < 0.01, R^2^ = 0.439). The data were obtained using the logistic regression analysis. (**D**) A schematic model of CASC2 functioning as a decoy by competitively binding miR-18a, upregulating the specific repressor *PIAS3* of STAT3 signaling, and suppressing colorectal carcinogenesis.

To further demonstrate the existence of specific interplay among CASC2, miR-18a and *PIAS3*, we monitored both mRNA and protein levels of *PIAS3* and STAT3-mediated downstream target genes in knockdown of CASC2 CRC cells. We utilized siRNA against CASC2 and examined the effect of CASC2 on *PIAS3* and STAT3-mediated downstream target genes expression. As shown in Fig. 5A,B, knockdown of CASC2 by siRNA triggered a significant silencing effect on *PIAS3*, whereas the expression levels of STAT3, pSTAT3 and c-Myc were dramatically up-regulated in response to the down-regulation of CASC2.

Furthermore, when we co-transfected psiCHECK2-CASC2-WT with miR-18a and RLuc-*PIAS3*-WT luciferase reporters, overexpression of CASC2 partially restored the luciferase activity of miR-18a-mediated suppression (Fig. 4C,D). Additionally, in the above CRC samples, the expression of CASC2 was significantly correlated with the expression of *PIAS3* (*P* < 0.01, R^2^ = 0.439; Fig. 5C). Together, these data indicate that CASC2 can function as a ceRNA by competitively binding miR-18a, thereby relieving the suppression of *PIAS3* expression by miR-18a in CRC.

## Discussion

The noncoding portion of the genome accounts for greater than 90% of the total mammalian genome. Studies have demonstrated that among these ncRNAs, ~18% of lncRNAs are associated with human tumors, compared with only 9% of human protein-coding genes^36^, suggesting that lncRNAs could act as major contributors to carcinogenesis and cancer progression. Thus, the roles of dysregulated lncRNAs in the pathogenesis of most cancers have garnered increased scientific interest in recent years.

Accumulating evidence confirms that lncRNAs can perform biological functions in both trans and cis. For example, the well-characterized lncRNA, HOTAIR, can physically interact with chromatin-remodeling complexes and recruits these complexes to specific genomic DNA sequences, thereby affecting the transcription regulation of specific genes^37^. Moreover, perturbation experiments demonstrate that some lncRNAs act as cis-regulators that exert function on transcripts in their immediate genomic neighborhood, as proposed for lincRNA-21^38^. It has recently been discovered that some lncRNAs can serve as miRNA “sponges” by sharing common MREs. These interactions influence post-transcriptional regulation by inhibiting available miRNA activity.

In the present study, we investigated the potential role of CASC2 as a ceRNA of the *PIAS3* gene by competing for miRNA-18a binding sites and thereby regulates the expression of the *PIAS3* mRNA targeted by this miRNA. Our study in clinical samples and CRC cell lines demonstrates that CASC2 is downregulated, and the downregulation of CASC2 is significantly associated with advanced pathological stage in patients with CRC. Overexpression of CASC2 is able to inhibit cell proliferation and tumor growth both *in vitro* and *in vivo* by extending G_0_/G_1_-S phase transition. These findings suggest that CASC2 plays a critical role in the modulation of CRC progression.

To fully understand CRC pathogenesis, we focused on the mechanism of CASC2 as a ceRNA from bioinformatics analysis. In support of this notion, qRT-PCR analysis showed that miR-18a expression is upregulated in the CRC tissues, and inversely correlates with CASC2 expression. Consistent with our findings, miR-18a has been well studied in several cancers with particularly high expression^31^,^32^,^39^,^40^. Furthermore, bioinformatics prediction combined with experimental analysis provides further support for the interaction of CASC2/miR-18a activity. The above results prompted us to investigate the miRNA-related functions of CASC2 in CRC pathogenesis. Recent work reported that overexpression of miR-18a could promote cell proliferation in gastric cancer by regulating *PIAS3* expression and consequentially increase STAT3 activity, leading to enhanced activation of genes downstream of STAT3 modulated by CASC2-targeting miRNAs. The importance of *PIAS3* has been well documented in a variety of human cancers^41^,^42^,^43^. *PIAS3* was originally identified as an endogenous transcriptional repressor of STAT3 signaling, and the dysregulated STAT3 activation was correlated with several malignant human diseases by regulating genes encoding anti-apoptotic and proliferation-associated proteins.

In the current study, luciferase and qRT-PCR assays also confirm that *PIAS3* is a direct target of miR-18a, and up-regulation of miR-18a enhances STAT3-mediated gene expression by regulating the expression of *PIAS3*. To further confirm the role of *PIAS3* as a miRNA-18a “sponge”, we observed that down-regulated CASC2 leads to decreased in *PIAS3* mRNA and protein levels, whereas overexpression of CASC2 restores *PIAS3* synthesis to high levels in CRC cells. This result reveals directly competitive binding with miR-18a between CASC2 and *PIAS3* mRNA, further indicating that CASC2 functions as a ceRNA and modulates the expression of miR-18a targets in CRC to suppress cancer progression (Fig. 5D).

In summary, we have demonstrated that CASC2 plays an important role in the pathobiology of human CRC by functioning as a ceRNA to regulate the expression of key genes. Therefore, the pleiotropic effects of CASC2 on CRC tumorigenesis suggest that CASC2 may potentially act as an effective therapeutic candidate for CRC.

## Methods

### Patient tissues and cell lines

CRC tissues and adjacent normal tissues (located >5cm away from the tumor border) in this study were collected from 68 CRC patients. All tissues were histopathologically confirmed and no patient had ever received any therapy before surgery. Written informed consent was obtained from all patients at recruitment. The clinical features of patients are listed in Table 1. All experiments in the study were performed in accordance with guidelines and regulations and were approved by the IRB (2015007) of First Affiliated Hospital of Wenzhou Medical University (Wenzhou, China).

### Cell lines

Human CRC cell lines (CACO2, SW480, SW620, HCT-116 and HT-29) were purchased from the American Type Culture Collection (USA). The human normal intestinal mucous cell line (CCC-HIE-2) was obtained from Type Culture Collection of the Chinese Academy of Medical Sciences (Beijing, China), and the human embryonic kidney 293 cell (HER293) were purchased from Institute of Biochemistry and Cell Biology of the Chinese Academy of Sciences (Shanghai, China).

### Quantitative real-time PCR (qRT-PCR) analysis

Total RNA was extracted from each cell sample and fresh frozen CRC tissues using Trizol reagent (Life Technologies, Carlsbad, CA, USA). The absorbance ratio at 260/280 nm of the isolated RNA were all over 2.0 measured with NanoDrop ND-1000 spectrophotometer. First-strand cDNA was synthesized by M-MLV reverse transcriptase (Invitrogen). The expression of CACS2 mRNA from tissue samples or the cultured cells was quantified according to the manufacturer’s instructions using SYBR Premix EX Taq™ II kit (TaKaRa, RR820A) on the ABI Prism 7500 (Applied Bio systems, Foster City, CA, USA). miR-18a miRNA was harvested using the PureLink™ miRNA Isolation Kit (Invitrogen, CA, USA) and miRNA expression was quantified by TaqMan MicroRNA Assay Kit (Applied Biosystems, Foster City, CA, USA). The relative expression levels of mRNA/lncRNA and miRNA were determined using the 2^−ΔΔCT^ method with human *GAPDH* and *U6* snRNA as internal controls, respectively. The sequences of the primers used in the study are shown in Table 2. Each experiment was performed in triplicate.

### Protein analysis

Western blot analyses were performed using standard methods as described previously^44^. Briefly, total proteins were extracted from cells by using the protein extraction buffer (Nova gen, Madison, WI, USA). Primary antibodies against the following proteins were used: PISA3 (Santa Cruz biotechnology, USA; 1:2000 dilution), STAT3 (Cell Signaling Technology, MA, USA; 1:1000 dilution), Survivin (Santa Cruz biotechnology, USA; 1:1000 dilution), pSTAT3 (Cell Signaling Technology, MA, USA; 1:1000 dilution), c-MYC (Abcam, Cambridge, UK; 1:5000 dilution), β-actin (Santa Cruz biotechnology, USA; 1:3000 dilution). Identical quantities of proteins were run on 10% SDS-polyacrylamide gel electrophoresis (SDS-PAGE) and transferred to polyvinylidene difluoride membranes (PVDF) (Millipore, Billerica, MA, USA). Protein bands were detected using the enhanced chemiluminescence (Pierce Biotechnology, Rockford, IL, USA) with imaging system (Bio-Rad, CA, USA). β-actin was used as a loading control.

### Construct generation and transient transfection

To study the effects of CASC2 on cell activity, plasmid complementary DNA lncRNA-CASC2 cDNA was constructed by introducing the cDNA sequence of CASC2 into the pcDNA3.1 expression vector (Invitrogen, Shanghai, China). The miRNA mimics, miRNA inhibitors and siRNAs for the knockdown of CASC2 expression were from GenePharma (Shanghai, China). For the transfection of the pcDNA-CASC2, miRNA mimics, miRNA inhibitors, and siRNAs, CRC cell lines (2 × 10^5^) were transfected with miRNA mimics, miRNA inhibitors or siRNAs at a final concentration of 25 nmol/l using Lipofectamine 2000 Reagent (Life Technologies). The above-mentioned cells were transfected with pcDNA-CASC2 constructs at a final concentration of 1 μg/μl according the protocol recommended by the manufacturer. After transfection for 48 h, total RNA from the harvested cells was isolated with Trizol reagent (Invitrogen, Carlsbad, CA, USA). The empty pcDNA3.1 vector and scramble sequence of miRNA mimics, miRNA inhibitors or siRNAs were used as the negative controls (NC).

### Luciferase reporter assay

Two luciferase reporters containing the wild type CASC2 (psiCHECK2-CASC2-WT) or mutant CASC2 were generated to analyze the interaction between CASC2 and miR-18a. Mutant CASC2 contained a mutation site (psiCHECK2-CASC2-MU) abolishing targeting by miR-18a. Colorectal cancer cells were cotransfected with 500 ng of the luciferase construct along with miR-18a mimics, negative mimics control, miR-18a inhibitors or negative control inhibitors. At 48 h post-transfection, luciferase activity assays were performed with the dual-luciferase reporter assay system.

### Cell proliferation assay *in vitro*

CRC cells (2000 cells per well) transfected with pcDNA-CASC2 or with negative control as described above were plated in 96-well plates. Cell proliferation was assessed using CCK-8 assay kits (Dojindo Laboratory, Kumamoto, Japan) daily over four consecutive days (1, 2, 3 and 4 day) according to the manufacturer’s protocol. For colony formation experiments, 200 transfected cells were plated in six-well plates for approximately 2 weeks at 37 °C in a 5% CO2 incubator. Colony formation was determined by counting the number of visible colonies after staining with 0.1% crystal violet (Sigma, USA).

### Cell cycle analysis

For cell cycle analysis, after the transfected cells described above seeded on six-well plates for 48 h, the cells were collected by centrifugation and then fixed with 70% ethanol at 4 °C overnight. Finally, cellular DNA content was analyzed by flow cytometry (FACScalibur; BD Biosciences) after propidium iodide (PI) staining.

### Tumor formation assay in nude mice

4–5 weeks old female BALB/c nude mice were maintained in pathogen-free conditions in accordance with the national guidelines of the Institutional Animal Care and Use Committee and were approved by the Shanghai Laboratory Animal Center at the Chinese Academy of Sciences (Shanghai, China). For the xenograft experiments, 24 nude mice were randomly divided into four groups of equal size (six per group) (pcDNA-CASC2-CACO2 group, empty vector-CACO2 group, pcDNA-CASC2-HT-29 group, empty vector-HT-29 group). Transfected cells were suspended in physiological saline, and a volume of 100 μl cell suspension containing 6 × 10^6^ cells was injected subcutaneously into the posterior flank of each nude mice (6 mice/group). When a tumor was palpable, tumor growth was measured every 2 days with calipers, and tumors volume was calculated as length × width^2^ × 0.5.

### Statistical analysis

All data are presented as the mean ± standard deviation (SD) from three independent experiments with triplicate samples. Statistical analyses were performed with SPSS version 17.0 software and GraphPad Prism 5.0. ANOVA analysis, chi-squared test, and paired *t*-tests were performed for statistical comparisons as appropriate. All *P*_values_ were two-sided, and a *P*_value_ < 0.05 was considered significant.

## Additional Information

**How to cite this article**: Huang, G. *et al.* The long noncoding RNA CASC2 functions as a competing endogenous RNA by sponging miR-18a in colorectal cancer. *Sci. Rep.* **6**, 26524; doi: 10.1038/srep26524 (2016).

## Supplementary Material

Supplementary Figure 1

## Figures and Tables

**Table 1:** Relationship between CASC2 and clinicopathological parameters in colorectal cancer patients (N = 68).

Characteristics	Expression of CASC2		*P*_value_^a^
	Low-CASC2 group	High-CASC2 group	
Sex			
male	15	20	
female	19	14	0.225
Gender			
<60	16	19	
≥60	18	15	0.467
Lymph node metastasis			
N0	13	16	
N1	11	9	
N2	10	9	0.561
Tumor size			
<2 cm	19	23	
≥2 cm	15	11	0.318
TNM stages			
I + II	11	20	
III + IV	23	14	**0**.**028**^b^
Histological type-differentiation			
Well	13	18	
moderate	12	10	
Poor	9	6	0.221

^a^Chi-square test.

^b^*P* < 0.05.

**Table 2:** Primers sequences used for expression analysis.

Gene	Primers sequence	
CASC2	Forward	5′-gctgatcagagcacattgga-3′
	Reverse	5′-ataaaggtggccacaactgc-3′
PIAS3	Forward	5′-gaagcgcactttacctttgc-3′
	Reverse	5′-gcacagtttcccattgacct-3′
STAT3	Forward	5′-ctggcctttggtgttgaaat-3′
	Reverse	5′-aaggcacccacagaaacaac-3′
Survivin	Forward	5′-acctgaaagcttcctcgaca-3′
	Reverse	5′-taacctgccattggaacctc-3′
c-Myc	Forward	5′-ttcgggtagtggaaaaccag-3′
	Reverse	5′-agcagctcgaatttcttcca-3′
GAPDH	Forward	5′-aggggagattcagtgtggtg-3′
	Reverse	5′-cgaccactttgtcaagctca-3′
