# Supplementary material for: The long noncoding RNA CASC2 functions as a competing endogenous RNA by sponging miR-18a in colorectal cancer
Source: Sci Rep. 2016 May 20;6:26524. doi: 10.1038/srep26524 (PMC4873821; doi:10.1038/srep26524)

Long noncoding RNA CASC2 functions as a competing endogenous RNA by sponging miR-18a in colorectal cancer

Guangli Huang, Xiaoli Wu, Shi Li, Xiaoqun Xu, Hua Zhu, Xiangjian Chen

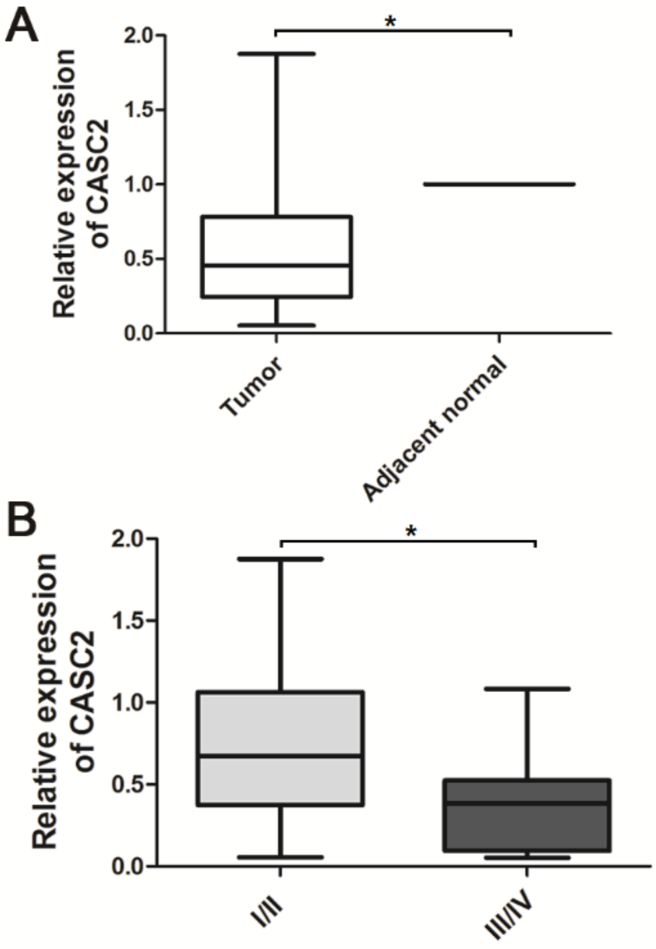

Supplement: Supplementary Figure 1 [file srep26524-s1.pdf]
